# Molecular Characterization of a Fus3/Kss1 Type MAPK from *Puccinia striiformis* f. sp. *tritici*, PsMAPK1

**DOI:** 10.1371/journal.pone.0021895

**Published:** 2011-07-14

**Authors:** Jun Guo, Xiwei Dai, Jin-Rong Xu, Yulin Wang, Pengfei Bai, Furong Liu, Yinghui Duan, Hong Zhang, Lili Huang, Zhensheng Kang

**Affiliations:** 1 State Key Laboratory of Crop Stress Biology for Arid Areas and College of Plant Protection, Northwest A&F University, Yangling, Shaanxi, People's Republic of China; 2 State Key Laboratory of Crop Stress Biology for Arid Areas and College of Life Science, Northwest A&F University, Yangling, Shaanxi, People's Republic of China; 3 Department of Botany and Plant Pathology, Purdue University, West Lafayette, Indiana, United States of America; Seoul National University, Korea, Republic of

## Abstract

*Puccinia striiformis* f. sp. *tritici* (*Pst*) is an obligate biotrophic fungus that causes the destructive wheat stripe rust disease worldwide. Due to the lack of reliable transformation and gene disruption method, knowledge about the function of *Pst* genes involved in pathogenesis is limited. Mitogen-activated protein kinase (MAPK) genes have been shown in a number of plant pathogenic fungi to play critical roles in regulating various infection processes. In the present study, we identified and characterized the first MAPK gene *PsMAPK1* in *Pst.* Phylogenetic analysis indicated that *PsMAPK1* is a YERK1 MAP kinase belonging to the Fus3/Kss1 class. Single nucleotide polymerphisms (SNPs) and insertion/deletion were detected in the coding region of *PsMAPK1* among six *Pst* isolates. Real-time RT-PCR analyses revealed that *PsMAPK1* expression was induced at early infection stages and peaked during haustorium formation. When expressed in *Fusarium graminearum*, *PsMAPK1* partially rescued the *map1* mutant in vegetative growth and pathogenicity. It also partially complemented the defects of the *Magnaporthe oryzae pmk1* mutant in appressorium formation and plant infection. These results suggest that *F. graminearum* and *M. oryzae* can be used as surrogate systems for functional analysis of well-conserved *Pst* genes and *PsMAPK1* may play a role in the regulation of plant penetration and infectious growth in *Pst*.

## Introduction

In a variety of eukaryotic organisms, a family of serine/threonine protein kinases known as the mitogen-activated protein kinases (MAPKs) play critical roles in the transduction of a variety of extracellular signals and regulation of various development and differentiation processes [1]. The MAPK cascades are conserved in eukaryotes and have been studied extensively in many organisms. In filamentous fungi, MAPKs mainly fall into three subgroups represented by Fus3/Kss1, Slt2, and Hog1 of *Saccharomyces cerevisiae* [2], [3], [4]. The Fus3/Kss1 homolog is more extensively studied than the other two MAPKs in fungal pathogens [1], [5], [6], [7]. In the rice blast fungus *Magnaporthe oryzae*, the *PMK1* MAP kinase gene is essential for appressorium formation and invasive growth [8]. In the wheat scab fungus *Fusarium graminearum*, the *map1* deletion mutants are female sterile, non-pathogenic, and reduced in conidiation and infectious growth [9], [10]. In *Ustilago maydis*, Kpp2 (Ubc3) and Kpp6 are two Fus3/Kss1 MAPKs with overlapping functions in mating and plant infection [11], [12]. The *ubc3*/*kpp2* mutant is defective in pheromone responses and the formation of filamentous dikaryons and reduced in virulence. In contrast, Kpp6 plays a more critical role in appressorial penetration than Kpp2. The *kpp6* mutant is reduced in virulence and defective in the penetration of plant cuticle [13]. The *kpp2 kpp6* double mutants are abolished in mating and nonpathogenic on maize plants. The Fus3/Kss1 homologs also have been functionally characterized in several human pathogens. In *Candida albicans*, the Cek1 MAPK plays a critical role in pathogenesis [14]. In *Cryptococcus neoformans*, the *CPK1* MAPK pathway is important for mating and haploid fruiting but dispensable for virulence [15].

Wheat stripe rust, caused by *Puccinia striiformis* f. sp. *tritici* (*Pst*), is one of the most important diseases of wheat worldwide. *Pst* is an obligate biotrophic fungus belonging to the Uredinales. The major phase of the stripe rust life cycle is urediniospores, which can germinate in water but germ tubes will die without host cells [16]. After successful adhesion to the wheat leaves, urediniospores produce germ tubes, which elongate along leaf veins until they encounter stomatal opening. After entering the substomatal space in wheat leaves, the fungus starts to successively differentiate other infection structures, e.g., substomatal vesicles, infection hypha, haustorial mother cell, and eventually haustoria, a structure to withdraw nutrients from host cells [17], [18], [19]. The majority of germ tubes penetrates stomata after 12 hours of germination, and formation of haustorial mother cells increases rapidly after 18 hours of inoculation [20]. During *Pst* infection, it is believed that the fungus recognizes various signals from the host plant at different stages and responds accordingly to establish a successful colonization. However, little is known about the role of signal transduction pathways in *Pst* and other rust fungi due to their obligate nature and the lack of an efficient and reliable transformation system. When expressed in *Ustilago maydis*, the *PtMAPK1* MAPK gene of *Puccinia triticina* was able to partially complement the *kpp2 kpp6* mutant for mating, virulence, and pathogenicity [21].

In a previous study [22], a MAPK gene, designated *PsMAPK1*, was identified in ESTs generated from a *Pst* cDNA library. Here we examined the expression profiles of *PsMAPK1* and its activities in two ascomycetous pathogens. *PsMAPK1* could partially complement the *F. graminearum map1* and *M. oryzae pmk1* mutants. Results from these studies indicate that *F. graminearum* or *M. oryzae* can be used as a heterologous expression system for functional studies with *Pst* genes and *PsMAPK1* may play an important role in regulating penetration and infectious growth of the wheat stripe rust fungus.

## Results

### *PsMAPK1* encodes a Fus3/Kss1 type MAP kinase

One of the expressed sequence tags (ESTs), 20C8, from a full-length cDNA library of *Pst* [22] is highly similar to *PgMAPK* from *P. graminis* f. sp. *tritici* (GenBank accession number EFP88010) and *PtMAPK1* from *P. triticina* (GenBank accession number AAY89655). By further sequencing analysis with clone 20C8, we designed primers and obtained the full-length cDNA of this MAPK gene from Chinese *Pst* race CYR32, which was designated *PsMAPK1* in this study (GenBank accession number HM535614). The open reading frame (ORF) of *PsMAPK1* was predicted to encode a 408-amino acid protein with typical features of MAP kinases, including 11 protein kinase domains and the TEY dual phosphorylation site (Fig. S1).

**Figure 1:**
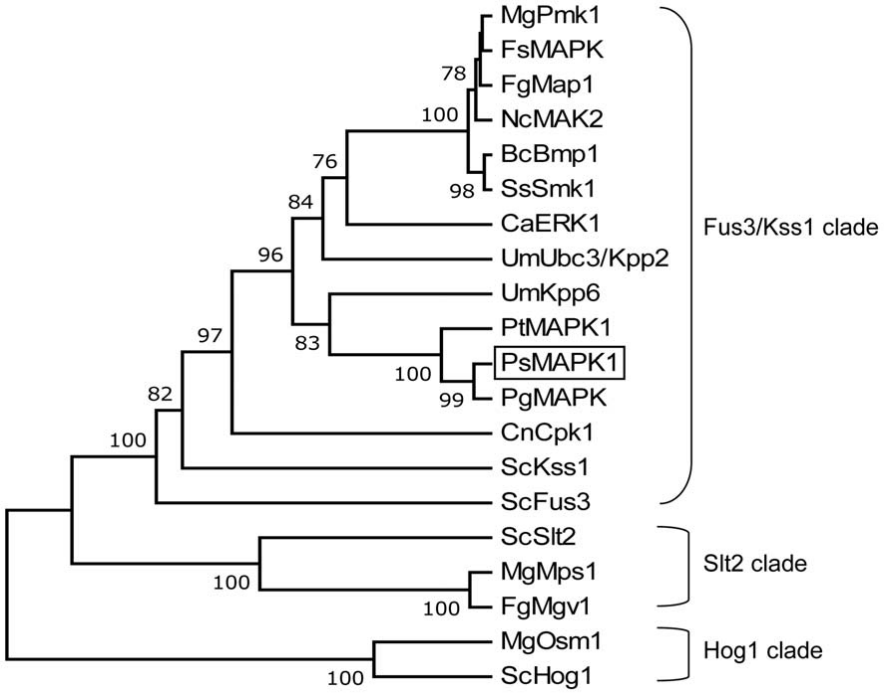
Phylogenetic analysis with PsMAPK1 and selected fungal MAP kinases (GenBank accession numbers in parenthesis). *Botrytis cinerea*, BcBmp1 (AAG23132); *Candida albicans*, CaERK1 (P28869); *Cryptococcus neoformans*, CnCpk1 (Q8NK05); *Fusarium solani*, FsMAPK (AAB72017); *Fusarium graminearum*, FgMap1 (AAL73403) and FgMgv1 (AAM13670); *Magnaporthe oryzae*, MgPmk1 (AAC49521), MgMps1 (AAC63682) and MgOsm1 (AAF09475); *Neurospora crassa*, NcMAK2 (AAK25816); *Puccinia striiformis* f. sp. *tritici*, PsMAPK1 (HM535614); *Puccinia triticina*, PtMAPK1 (AAY89655); *Puccinia graminis* f. sp. *tritici*, PgMAPK (EFP88010); *Saccharomyces cerevisiae*, ScFus3 (CAA49292), ScHog1 (CAA97680), ScKss1 (CAA97038) and ScSlt2 (CAA41954); *Sclerotinia sclerotiorum*, SsSmk1 (AAQ54908); *Ustilago maydis*, UmKpp6 (CAD43731) and UmUbc3/Kpp2 (AAF09452). The unrooted phylogram was constructed based on NJ analysis. Confidence of groupings was estimated by using 1,000 bootstrap replicates. Numbers next to the branching point indicate the percentage of replicates supporting each branch.

The level of conservation among the PsMAPK1 protein, PgMAPK from *P. graminis* f. sp. *tricitici*, PtMAPK1 from *P. triticina*, Pmk1 from *M. oryzae*, and *F. graminearum* Map1 is indicated in Figure S1. PsMAPK1 had the highest homology with PgMAPK (91% identity), followed by PtMAPK1 (87% identity), *M. oryzae* Pmk1 (75% identity) and *F. graminearum* Map1 (75% identity). Phylogenetic analysis revealed that PsMAPK1 is more closely related to *M. oryzae* Pmk1 and *F. graminearum* Map1 than to MAPKs belonging to the Slt2 and Hog1 (Fig. 1). In the Fus3/Kss1 clade, PsMAPK1 is more similar to MAPKs from basidiomycetous fungi than to those from ascomycetous fungi. Grouping of PsMAPK1 in the Fus3/Kss1 clade implies that it may play a role in mating, morphogenesis, or pathogenic development in *Pst* based on the functions of its orthologs in other plant pathogenic fungi [1], [8], [9], [10], [13].

To identify coding single nucleotide polymorphism (cSNP) in *PsMAPK1*, we performed PCR amplifications with cDNA of Chinese *Pst* races CYR32, CYR23, CYR25, CYR29, CYR31, and CYR33 (Table 1). At least six positive amplicons amplified with primers PKO1 and PKO2 were cloned and sequenced. In comparison with the *PsMAPK1* sequence from race CYR32, a total of 32 cSNPs, including 27 synonymous cSNPs, five non-synonymous cSNPs, and one amino acid insertion/deletion, were observed among *Pst* reference isolates (Table S1). Regarding five non-synonymous cSNPs, two were identified in CYR23 and three in CYR29. The one amino acid insertion/deletion was found in CYR29 (Fig. S2). These results indicate that although overall it is highly conserved, sequence variations do exist in the *PsMAPK1* gene among different isolates of *Pst* [2].

**Table 1:** Primers and strains used in the study.

Primers	Sequences (5′→3′)
FP1s	TTTTAGCCTGCCCATCCC
FP1as	CCCAGCACCTCCAGAATCA
FP2s	GGGACAGAGGGTATTGTTTTG
FP2as	ATCCTGGCCGAGATGCTT
PMKO1	ATGGCAGCCGTTGTAGCTCT
PMKO2	TTAAGCAGTGGAGTGGAAGCT
CFg1	CCATCGATGGCAGCCGTTGTAGCTCTAC a
CFg2	CGCGGATCCTTAAGCAGTGGAGTGGAA ^b^
CMo1	CAGATCTTGGCTTTCGTAGGAACCCAATCTTCAATGGCAGCCGTTGTAGCTC
CMo2	CACCACCCCGGTGAACAGCTCCTCGCCCTTGCTCACAGCAGTGGAGTGGAAGCT
PKQF	CGCCTCTACTACTTCAACTACTGCC
PKQR	CCCTCGCCGATAACATCCATAAC
PsEFQF	TTCGCCGTCCGTGATATGAGACAA
PsEFQR	ATGCGTATCATGGTGGTGGAGTGA

Strains	Genotype/Comment
Guy11	A wide-type strain of *Magnaporthe oryzae*
nn78	The *pmk1* deletion mutant of Guy11
CM-10	nn78 complemented with RP27 promoter :: *PsMAPK1-eGFP*
PH-1	A wide-type strain of *Fusarium graminearum*
*Δmap1*	The *map1* deletion mutant of PH-1
CF-6	*Δmap1* complemented with *trpC* promoter :: *PsMAPK1*
CF-8	*Δmap1* complemented with *trpC* promoter :: *PsMAPK1*
CF-10	*Δmap1* complemented with *trpC* promoter :: *PsMAPK1*

a The *Cla*I and *Bam*HI sites introduced in the primer sequences were underlined.

### *PsMAPK1* expression was increased during haustorium development

**Figure 2:**
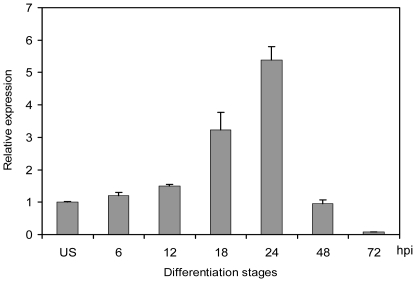
Assays for the transcript levels of *PsMAPK1* during different infection stages. RNA samples were isolated from urediniospores or leaves of wheat cultivar Suwon 11 inoculated with CYR32 and collected at the indicated time points. The expression level of *PsMAPK1* was estimated by the comparative ΔΔCt method with the elongation factor gene of *Pst* as the endogenous reference for normalization. Relative quantification was computed with their expression levels in different stages in comparison to that in urediniospores. Means and standard errors were calculated from three biological replicates. US, urediniospores; hpi, hours post inoculation.

Histological observation with wheat plants infected by *Pst* [20] had showed that urediniospores germinated and began to produce the germ tube at 6 hours post-inoculation (hpi). A penetration hypha then entered the stomatal pore and formed a substomatal vesicle, which further differentiates into primary hyphae and haustorium mother cells at 12 hpi in infected wheat leaves. Primary haustoria appeared at 18 hpi and could be observed at most of the infection sites at 24 hpi. From 48 to 72 hpi, secondary hyphae were differentiated and grew rapidly in colonized plant tissues. In qRT-PCR assays with RNA isolated from different infection stages, we found that the transcript level of *PsMAPK1* gradually increased at early infection stages from 6 to 24 hpi but decreased after 24 hpi (Fig. 2). During early infection stages, from 6 to 12 hpi, *PsMAPK1* transcription was not significantly up-regulated. However, at 18 and 24 hpi, i.e., haustorial formation stage, the amount of *PsMAPK1* mRNA was increased over 3- to 5- fold in comparison with that in urediniospores. After that, the transcription of *PsMAPK1* was down-regulated during the secondary hypha development stage.

### *PsMAPK1* partially complements the *F. graminearum map1* mutant

**Figure 3:**
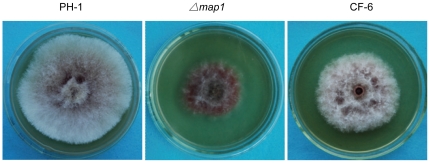
Colony morphology of *Fusarium graminearum* strains. Colonies of the wild-type (PH-1), *map1* deletion mutant, and complemented strain (CF-6) grown on PDA plates for 5 days.

To determine its function in *F. graminearum*, the *PsMAPK1* gene was cloned between the *Bam*HI and *Cla*I sites of pHZ100-TCH and transformed into protoplasts of the *F. graminearum map1* mutant [9], [10]. Ten resulting neomycin-resistant transformants were obtained and confirmed by PCR analysis to contain the transforming *PsMAPK1* construct. These transformants had identical phenotypes although only data with transformant CF-6 were presented below. The *F. graminearum map1* mutant has a reduced growth rate [10]. On PDA plates, colonies of transformant CF-6 were larger and more fluffy than those of the m*ap1* mutant (Fig. 3). The growth rate of transformant CF-6 measured at different time points was significantly increased in comparison with that of the *map1* mutant (P<0.05). However, CF-6 and other *map1*/*PsMAPK1* transformants still grew slower than the wide-type strain PH-1 (Fig. 3). These results indicate that expression of the *PsMAPK1* gene in *F. graminearum* partially complemented the defects of the *map1* mutant in vegetative growth.

**Figure 4:**
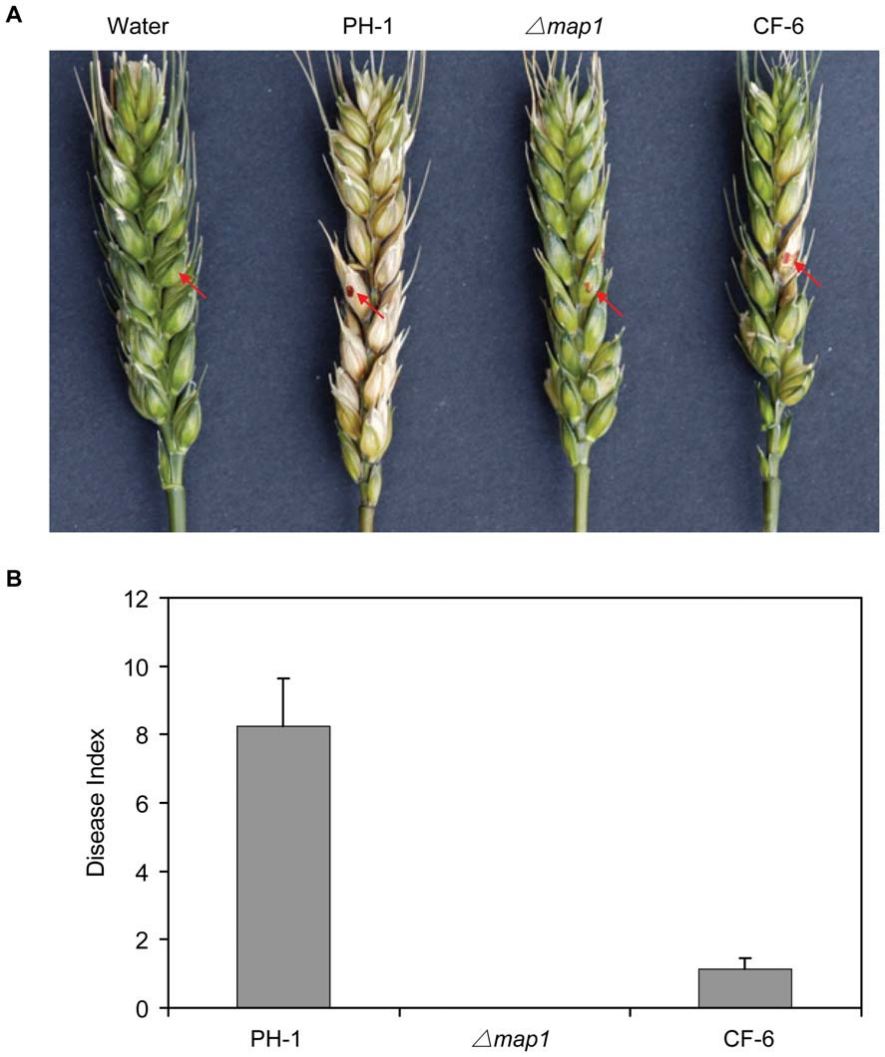
Infection assays with flowering wheat heads. (A) Wheat heads were drop-inoculated with sterile water or conidia from the wild-type strain PH-1, *map1* mutant, and complemented *map1*/PsMAPK1 transformant CF-6. The inoculation sites are marked with arrows. Typical heads were photographed 14 days after inoculation. (B) Disease index scores of PH-1, *map1*, and CF-6. Mean and standard error were calculated from three independent infection assays.

In *F. graminearum*, *MAP1* is essential to cause the wheat scab or head blight disease [10]. To determine the virulence of complemented strain CF-6, flowering wheat heads were point-inoculated with conidia suspensions. On wheat heads inoculated with the wild-type strain PH-1, typical scab symptoms were observed in the inoculated and neighbouring spikelets 14 days post-inoculation (dpi). No scab symptoms could be detected on wheat heads inoculated with the *map1* mutant (Fig. 4A). Under the same conditions, wheat heads inoculated with the complemented strain CF-6 developed scab symptoms at the inoculated spikelets (Fig. 4A). However, most of the spikelets adjacent to the inoculation sites remained healthy 14 dpi. Transformant CF-6 rarely (approximately 13%) spread from the inoculated spikelet and to neighbouring spikelets. On average, the complemented strain CF-6 had a disease index score of 1.1 (Fig. 4B), indicating that the defects of the *F. graminearum map1* mutant in plant infection also was partially complemented by the *PsMAPK1* gene. We repeated infection assays with two additional complementation strains CF-8 and CF-10 (Table 1) and obtained similar results (data not shown).

### Complementation of the *M. oryzae pmk1* deletion mutant by *PsMAPK1*

**Figure 5:**
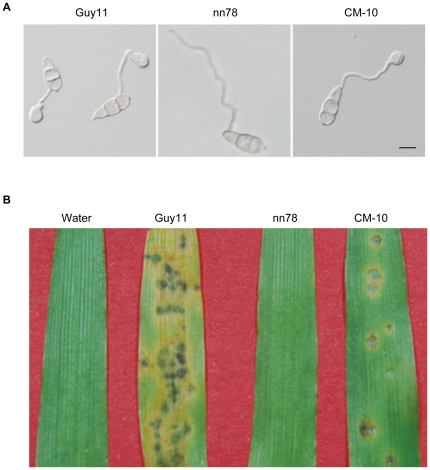
Complementation of the *pmk1* mutant with the GFP-PsMAPK1 fusion construct. (A) Appressorium formation assay. Germ tubes from the wild-type strain (Guy11) developed appressoria by 18 h, but no appressorium formation was observed in the *pmk1* mutant (nn78). Under the same conditions, a transformant of nn78 expressing the GFP-PsMAPK1 fusion construct (CM-10) formed appressoria. Bar = 25 µm. (B) Barley infection assay. Left to right, barley leaves were sprayed with sterile and conidia of Guy11, nn78, or CM-10. Typical leaves were photographed at 6 days post inoculation.

The PsMAPK1 protein shares 75% identity and 88% similarity with Pmk1 of *M. oryzae*. To test whether it can functionally complement the *pmk1* mutant, we transform the PsMAPK1-eGFP fusion construct (under the control of RP27 promoter) into the *pmk1* mutant nn78 [8]. Neomycin-resistant transformants were isolated and verified by PCR to confirm the introduction of *PsMAPK1*. One resulting *pmk1*/*PsMAPK1* transformant CM-10 was assayed for appressorium formation and plant infection. While over 90% of the germ tubes formed appressoria by 24 h in Guy11, the original *pmk1* mutant, nn78, failed to form appressoria under the same conditions (Fig. 5A). In transformant CM-10, approximately 25% of the germ tubes formed appressoria by 24 h (Fig. 5A). However, no GFP signals could be detected in appressoria formed by CM-10 (data not shown).

To determine whether the GFP-*PsMAPK1* construct could complement the defects of the *pmk1* mutant in plant infection, eight-day-old barley seedlings of cultivar NB6 were sprayed with conidia of transformant CM-10. At 6 dpi, leaves inoculated with CM-10 or Guy11 developed typical blast lesions (Fig. 5B). No lesions were observed on leaves sprayed with water or conidia of nn78 (Fig. 5B). In comparison with Guy11, transformant CM-10 caused fewer and smaller lesions on barley leaves. These results indicated that *PsMAPK1* could partially complement the *pmk1* mutant in appressorium formation and plant infection.

## Discussion

In this study we described the isolation and characterization of the first MAPK gene from the wheat stripe rust fungus *Pst*. Phylogenetic analysis revealed *PsMAPK1* is more closely related to Fus/Kss1 orthologs from basidiomycetes than those from ascomycetes. In the corn smut fungus *U. maydis*, Ubc3/Kpp2 and Kpp6 are two MAP kinase genes belonging to the Fus3/Kss1 (YERK1) clade. Ubc3/Kpp2 and Kpp6 have overlapping functions in mating and plant infection but Kpp6 plays a more critical role in appressorial penetration than Kpp2 [11], [12]. Other basidiomycetes, including *Cryptococcus neoformans* and *Phanerochaete chrysosporium* [7], [13], also have two YERK1 MAPK genes. In the genome sequence of *P. graminis* f. sp. *tritici*, we also identified two Fus3/Kss1 MAPK homologs (GenBank accession numbers EFP88010 and EFP80661). Similar to *PsMAPK1*, EFP88010 of *P. graminis* f. sp. *tritici* has six introns. No introns were found in EFP80661. These observations were consistent with what has been reported in *P. triticina* and *U. maydis* MAPKs [13], [21]. Based on the sequence similarity and intron distribution pattern, we conclude that PsMAPK1 is more closely related to Kpp6 than to Ubc3/Kpp2 of *U. maydis*. It is likely that, similar to *P. triticina* and *P. graminis* f. sp. *tritici*, *Pst* has two YERK1 genes. As Kpp6 in *U. maydis*, *PsMAPK1* may play a more important role in early stages of infection in *Pst*, such as haustorium formation, than the other Fus3/Kss1 homolog, which may be involved in stabilization and maintenance of the dikaryotic state [21].

Single nucleotide polymorphism markers are important tools for various studies, such as recombination, chromosomal dynamics, genome rearrangement, and genetic relatedness between individuals. SNPs can be located in the coding or intergenic regions [23], [24]. In coding regions, SNPs may result changes in protein structures and functions [25]. In this study, we identified SNPs in the *PsMAPK1* coding region among different field isolates of *Pst*. In the human genome, SNPs are estimated to occur once every 1 kb [26]. In *Candida albicans*, the average SNP frequency set was 1 SNP per 83 bp [27]. Between the *PsMAPK1* sequences from *Pst* isolates CYR29 and CYR32, the SNP frequency was about 1 SNP per 50 bp, indicating that significant genetic variations exist among different isolates of *Pst*. In a previous study, a higher genetic recombination rate was observed in strains originating from the Tianshui county, suggesting the existence of sexual reproduction in *Pst* [28]. A recent study also revealed that *Berberis* can serve as the alternate host for *Pst*. Therefore, we speculate that sexual recombination may account for the high gene variations in *Pst*. In addition, the SNPs identified from *Pst* reference isolates can be used as useful molecular markers to distinguish different races in the field.

In a number of plant pathogenic fungi, Fus3/Kss1 orthologs have been shown to regulate various plant infection processes, such as appressorium formation in *M. oryzae* [8], *Colletotrichum lagenarium* [29], and *Cochliobolus heterostrophus* [30]. In the barley powdery mildew fungus *Blumeria graminis*, a MAPK gene also has been implicated in the regulation of appressorium development by complementation assays [31]. In this study, we found that *PsMAPK1* could partially complement the *pmk1* mutant in appressorium formation and plant infection, indicating the functional conservation between *PsMAPK1* and *PMK1*. Real-time RT-PCR assays revealed that *PsMAPK1* has an increased expression level in early plant infection stages. Its expression peaked during haustorium formation, which is similar to the expression pattern of *PtMAPK1* in *P. triticina* during plant infection [21]. These observations suggest that the development of highly specialized infection structures such as haustoria in rust fungi is regulated by a well conserved MAPK signaling cascade.

Expression of the *PsMAPK1* gene also partially restored the defects of the *F. graminearum map1* mutant in vegetative growth and plant infection. The fact that the YERK1 subfamily genes are highly conserved may explain for observed functional relatedness among pathogens with different plant infection mechanisms, such as *F. graminearum*, *M. oryzae*, and *Pst*. However, the phenotypes of the *map1* and *pmk1* mutants were only partially complemented, indicating that *PsMAPK1* is not fully functional in ascomycetous fungi. *Pst* is a rust pathogen that has a distinct life style from *M. oryzae* and *F. graminearum*. During evolution, sequence and structural changes in *PsMAPK1* may enable it to interact with other components of this MAPK pathway that are not conserved. These changes may reduce the efficiency of *PsMAPK1* in signal transduction in ascomycetes and account for partial complementation. Complementation assays with the MAPK mutants of the basidiomycetous pathogen *U. maydis* may be better for functional analysis with *PsMAPK1*. However, the *PtMAPK1* gene from *P. triticina* also only partially complemented the *U. maydis kpp2* mutant [21]. Sequence alignment revealed that PsMAPK1 shares 77%, 74%, 75%, and 75% amino acid sequence identity with Kpp6 and Kpp2 of *U. maydis*, Pmk1 of *M. oryzae*, and Map1 of *F. graminearum*, respectively. Therefore, the overall homology of PsMAPK1 with its orthologs from *U. maydis* is not significantly higher than with its orthologs from two ascomycetes.

Although *PsMAPK1* partially rescued the *pmk1* mutant for appressorium formation, no GFP signals could be detected in appressoria formed by transformant CM-10. A similar observation has been reported by Yang and colleagues [32]. Although expression of a *COM1*-eGFP fusion construct complemented the *com1* deletion mutant, GFP signals were not detectable in vegetative hyphae, conidia, germination tubes, appressoria, or infection hyphae of *M. oryzae*. The abundance of the PsMAPK1-eGFP fusion proteins may be too low to be detected by fluorescence microscopy in these transformants. However, it is more likely that the PsMAPK1-eGFP fusion proteins are not stable or lack fluorescent signals. Fusion with the PsMAPK1 protein may change the structure of GFP proteins.

In qRT-PCR assays, *PsMAPK1* was highly expressed during the haustorium formation stage. However, its expression was not significantly up-regulated from 6 to 12 hpi, which corresponded to the appressorium formation stage. There are contradictory reports on the formation of appressoria by *Pst* in penetration of wheat stomata [20], [33]. When wheat cultivar Mingxian 169 was inoculated with twelve *Pst* isolates worldwide, eight of them formed appressoria on wheat leaves although the percentage of appressorium formation was less than 3.29% (unpublished data). Race CYR32 used for qRT-PCR assays in this study did not form appressoria. Therefore, appressorium formation appears to be dispensable for *Pst* infection. Our qRT-PCR data suggest that *PsMAPK1* is not important for penetration through stomata by directional growth of germ tubes. Also, penetration of mesophyll cells by haustorium mother cell in *Pst* is a process that is more similar to appressorium penetration in *M. oryzae*. It is likely that *PsMAPK1* plays a critical role in the regulation of penetration peg formation by the haustorium mother cell and differentiation of haustoria in plant cells in *Pst*. In addition, similar to symbiosis of *Tuber borchii*, *Pst* may use this MAPK pathway for its biotrophic growth *in planta* [34].

## Materials and Methods

### Strains and culture conditions

*Pst* strains CYR32, CYR23, CYR25, CYR29, CYR31 and CYR33 were inoculated and propagated on wheat cultivar Huixianhong as described previously [35]. Fresh urediniospores were harvested from infected wheat plants. For RNA isolation, wheat leaves of susceptible cultivar Suwon 11 inoculated with CYR32 urediniospores were harvested at 6, 12, 18, 24, 48, and 72 hpi.

The *F. graminearum* and *M. oryzae* strains used in this study (Table 1) were maintained at 25°C as described [8], [36]. Protoplast preparation and transformation of *F. graminearum* were performed as described [36], [37]. Complete medium (CM) with 250 µg/ml hygromycin B (Calbiochem) and 250 µg/ml geneticin (Sigma, St. Louis, MO) was used for selection of transformants. Transformation of the *M. oryzae pmk1* mutant was performed as described [8], [38]. Appressorium formation assays and plant infection were conducted as previous described [39], [40].

### Nucleic acid manipulations

Standard molecular biology procedures were performed as previously described [41]. Genomic DNA was isolated from urediniospores of *Pst* strain CYR32 [42]. Total RNA was isolated from urediniospores and infected wheat leaves according to established procedures [35]. DNaseI treatment was used to remove genomic DNA. First-strand cDNA was synthesized using the SMART™ reverse transcription Kit (Clontech) with pd(N)6 random primer (Takara) in the presence of recombinant RNasin ribonuclease inhibitor (Promega) according to the manufacturer's instruction. To assay coding region single nucleotide polymorphisms of *PsMAPK1*, *PsMAPK1* ORF was amplified from cDNA of different *Pst* isolates with the Pfu proofreading polymerase (Promega) with primers PKO1 and PKO2 (Table 1).

### Isolation and sequence analysis of *PsMAPK1*

The sequence of clone 20C8 of a full-length *Pst* cDNA library [22] was highly similar to several fungal MAPKs. To obtain the full-length cDNA of *PsMAPK1* from Chinese *Pst* race CYR32, two primer pairs FP1 (FP1s and FP1as) and FP2 (FP2s and FP2as) were designed (Table 1). The genomic region of the *PsMAPK1* gene was amplified with primers PKO1 and PKO2 (Table 1). DNA sequencing was performed with an ABI3130 Genetic Analyzer (Applied Biosystems, CA, USA) using the BigDye Terminator Cycle Sequence chemistry (Applied Biosystems, CA, USA). DNA sequences were analyzed with the DNASTAR (http://www.dnastar.com), BLAST (http://www.ncbi.nlm.nih.gov/blast/), and ORF Finder (http://www.ncbi.nlm.nih.gov/gorf/gorf.html) programs. ClustalW 1.83 [43] and DNAMAN6.0 (Lynnon BioSoft, USA) were used for sequence alignment analyses. MEGA4 [44] was used for phylogenetic analysis using the Neighbor-Joining (NJ) method. The *PsMAPK1* gene sequence has been deposited in GenBank (GenBank accession number HM535614).

### Quantitative RT-PCR (qRT-PCR)

To analyze the expression levels of *PsMAPK1*, relative quantification of gene expression was performed by using SYBR Green qRT-PCR mixtures in an ABI prism 7500 sequence detection system (Applied Biosystems, USA). PCR was performed with the program of 95°C for 1 min, and 40 cycles of 10 s at 95°C, 20 s at 60°C and 40 s at 72°C. The transcript level of *PsMAPK1* was calculated by the 2^−ΔΔCT^ method [45] with the *Pst* elongation factor 1 (EF1) gene [22] as the endogenous reference for normalization (Table 1). Transcript abundance was assessed with three independent biological replicates.

### Complementation of the *F. graminearum map1* mutant with *PsMAPK1*

For *F. graminearum* complementation assays, the vector pFgPsMAPK1 was constructed as follows. First, the *Spe*I-*Sal*I fragment containing the P_TrpC_-*hph-*T_TrpC_ cassette from pTFCM [46] was cloned into pHZ100 [47] as pHZ100-TCH. The open reading frame (ORF) of *PsMAPK1* was amplified with primers CFg1 and CFg2 (Table 1) and cloned between the *Bam*HI and *Cla*I sites of pHZ100-TCH to obtain the complementation construct pFgPsMAPK1. Plasmid pFgPsMAPK1 was transformed into the *map1* mutant as described [48]. The resulting neomycin-resistant transformants were confirmed by PCR analysis to contain the transforming vector pFgPsMAPK1.

Six-week-old plants of wheat cv. Xiaoyan 22 were used in infection assays with conidia collected from 5-day-old CMC cultures as previously described [36]. The third spikelet from the base of the inflorescence was injected with 10 µl of the conidium suspension (10^6^ conidia/ml). The inoculated wheat heads were covered with a small plastic bag for 2 days. Symptomatic spikelets in each head were counted 14 days after inoculation and disease index scores were calculated as described [49], [50] with results from three independent replicates. The raw data were subjected to an analysis of variance (ANOVA) followed by determining differences between treatment means by Duncan's multiple range test.

### Complementation of the *M. oryzae pmk1* mutant with *PsMAPK1*

For complementation assays with the *M. oryzae pmk1* mutant, the 1, 295 bp fragment of the *PsMAPK1* gene amplified with primers CMo1 and CMo2 (Table 1) was co-transformed with *Xho*I-digested pFL2 into *S. cerevisiae* strain XK1-25 [39]. Plasmid pMoPsMAPK1 containing the *PsMAPK1-eGFP* construct was transformed into protoplasts of the *pmk1* deletion mutant nn78 [8]. Zeocin-resistant transformants were isolated and verified by PCR with primers CMo1 and CMo2 to contain the *PsMAPK1* gene integrated in the *M. oryzae* genome. Appressorium formation and GFP signals were assayed as described [5], [51]. For plant infection assays, conidia were resuspended to 10^5^ conidia/ml in sterile distilled water. Eight-day-old barley seedlings of cultivar NB6 were used for spray infection assays as described previously [52], [53]. Lesion formation was examined 6 days after inoculation.

## Supporting Information

Figure S1 Sequence alignment of PsMAPK1 with *Puccinia triticina* PtMAPK1, *Puccinia graminis* f. sp. *tritici* PgMAPK, *Magnaporthe oryzae* Pmk1 and *Fusarium graminearum* Map1. Identical and similar residues are shaded in black and light grey, respectively. The 11 protein kinase subdomains are labeled with roman numerals on the top (Hanks et al. 1988). The tyrosine and threonine residues, two putative phosphorylation sites for MAP kinase, are indicated by asterisks.
(TIF)

Figure S2 Amino acid polymorphisms in the *PsMAPK1* gene of *Pst* isolates CYR32, CYR23 and CYR29. The arrow indicates a serine insertion in PsMAPK1 from CYR29. The five non-synonymous amino acid substitutions are shaded and marked with asterisks.
(TIF)

Table S1 Overview of the nucleotide variation in *PsMAPK1* from six *Pst* reference isolates.
(DOC)
