# Supplementary figures and images for: Molecular Characterization of a Fus3/Kss1 Type MAPK from Puccinia striiformis f. sp. tritici, PsMAPK1
Source: PLoS One. 2011 Jul 14;6(7):e21895. doi: 10.1371/journal.pone.0021895 (PMC3136484; doi:10.1371/journal.pone.0021895)

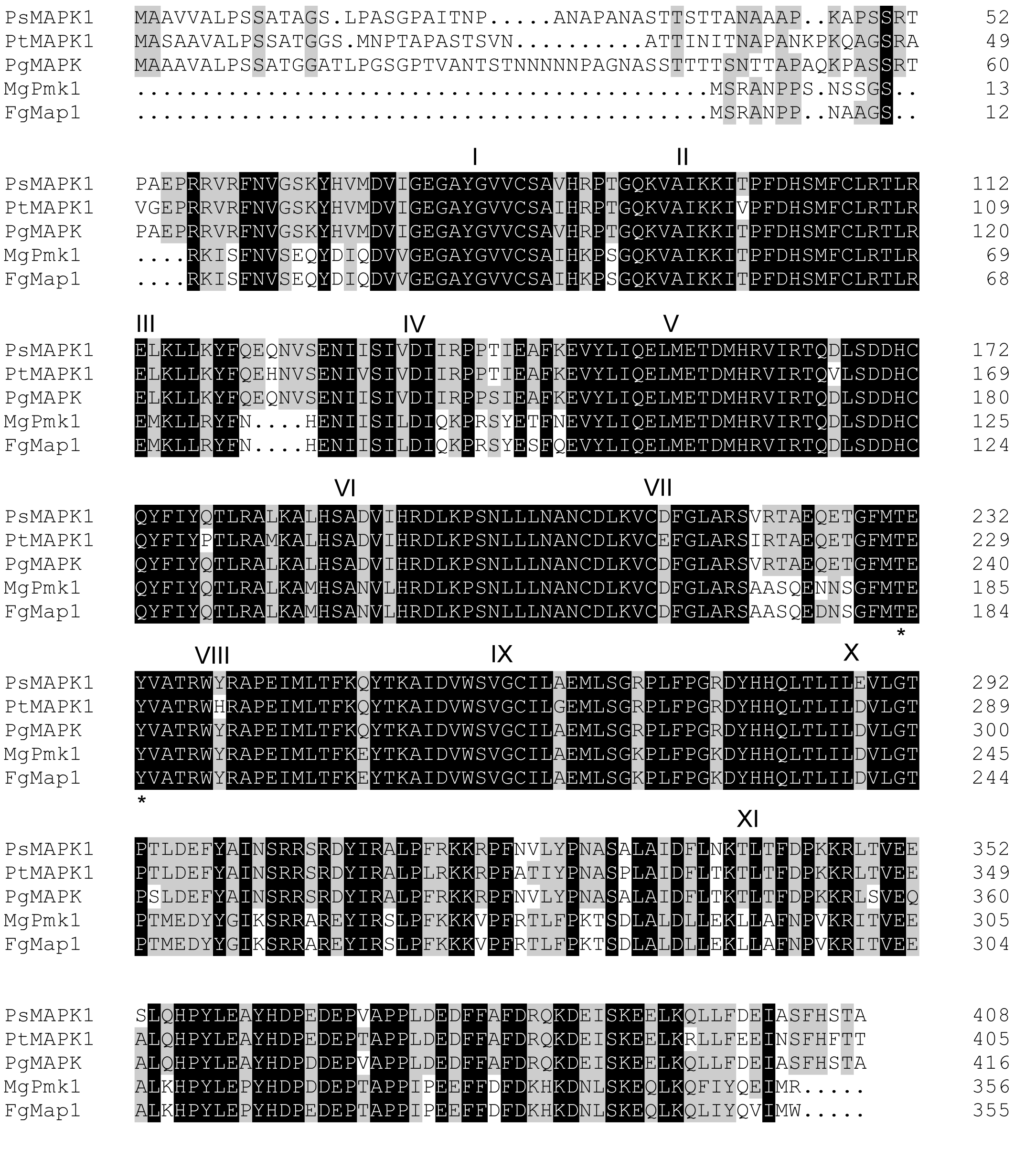

Supplement: Figure S1 — Sequence alignment of PsMAPK1 with Puccinia triticina PtMAPK1, Puccinia graminis f. sp. tritici PgMAPK, Magnaporthe oryzae Pmk1 and Fusarium graminearum Map1. Identical and similar residues are shaded in black and light grey, respectively. The 11 protein kinase subdomains are labeled with roman numerals on the top (Hanks et al. 1988). The tyrosine and threonine residues, two putative phosphorylation sites for MAP kinase, are indicated by asterisks. (TIF) [file pone.0021895.s001.tif]

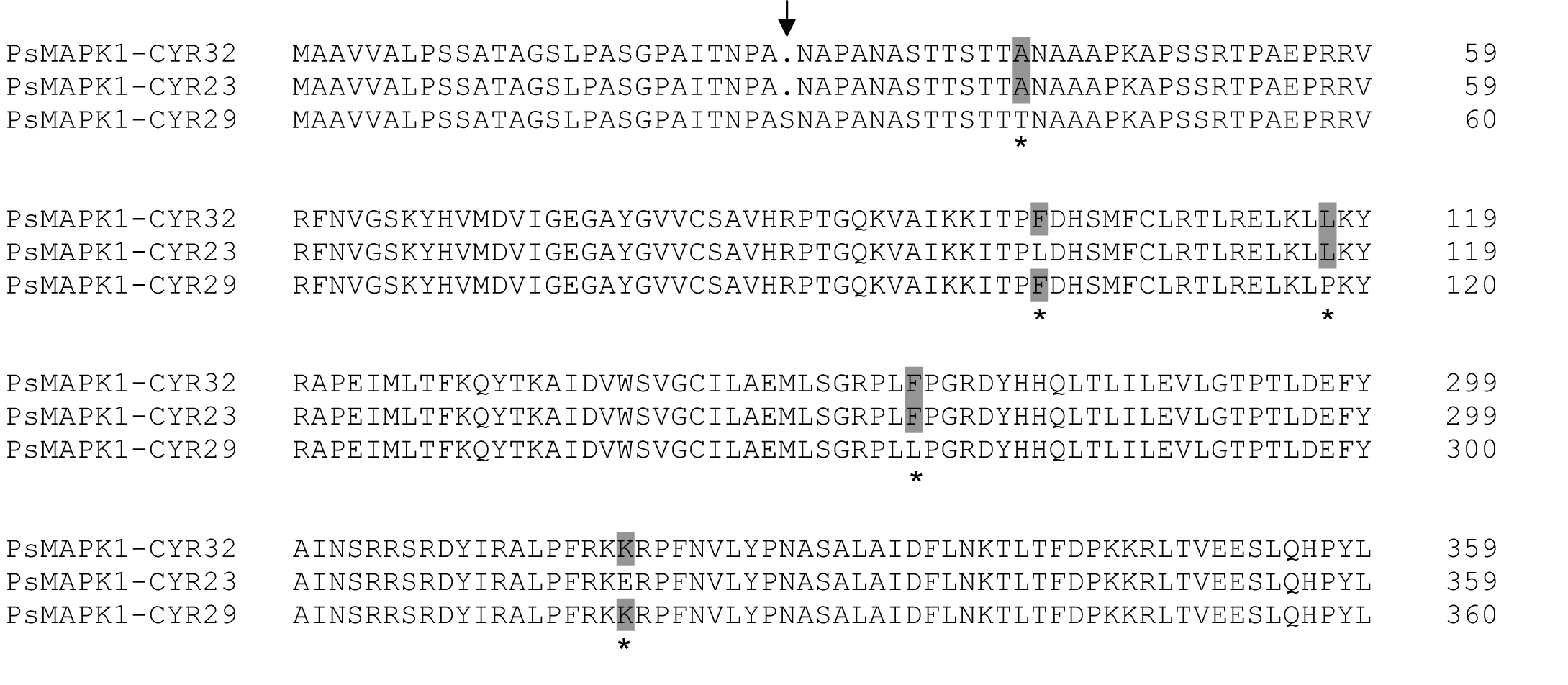

Supplement: Figure S2 — Amino acid polymorphisms in the PsMAPK1 gene of Pst isolates CYR32, CYR23 and CYR29. The arrow indicates a serine insertion in PsMAPK1 from CYR29. The five non-synonymous amino acid substitutions are shaded and marked with asterisks. (TIF) [file pone.0021895.s002.tif]
