# Supplementary material for: Molecular Characterization of a Fus3/Kss1 Type MAPK from Puccinia striiformis f. sp. tritici, PsMAPK1
Source: PLoS One. 2011 Jul 14;6(7):e21895. doi: 10.1371/journal.pone.0021895 (PMC3136484; doi:10.1371/journal.pone.0021895)
Supplement: Table S1 — Overview of the nucleotide variation in PsMAPK1 from six Pst reference isolates. (DOC) [file pone.0021895.s003.doc]

**Table S1.** Overview of the nucleotide variation in *PsMAPK1* from six *Pst* reference isolates.

| Nucleotide  Position**^a^** | Allele | | | | | |
| --- | --- | --- | --- | --- | --- | --- |
|  | CYR23 | CYR25 | CYR29 | CYR31 | CYR33 | CYR32 |
| 57 | C | C | A | C | C | C |
| 60 | T | T | A | T | T | T |
| 63 | C | C | T | C | C | C |
| 78 | T | T | C | T | T | T |
| 82 |  |  | +T |  |  |  |
| 83 |  |  | +C |  |  |  |
| 84 |  |  | +C |  |  |  |
| 96 | G | G | A | G | G | G |
| 120 | C | T | T | T | T | T |
| ^*^121 | G | G | A | G | G | G |
| 150 | T | T | C | T | T | T |
| 171 | C | C | T | C | C | C |
| 186 | C | C | T | C | C | C |
| 276 | A | A | G | A | A | A |
| ^*^304 | C | T | T | T | T | T |
| 312 | T | T | C | C | C | C |
| 333 | C | C | T | C | C | C |
| ^*^353 | T | T | C | T | T | T |
| 583 | C | C | T | C | C | C |
| 584 | C | C | A | C | C | C |
| 603 | C | C | C | T | C | T |
| 618 | C | T | T | T | T | T |
| 630 | G | G | C | G | G | G |
| 642 | T | T | C | T | T | T |
| 654 | G | G | T | G | G | G |
| 690 | C | C | T | C | C | C |
| ^*^823 | T | T | C | T | T | T |
| 837 | C | C | T | C | C | C |
| 885 | G | G | A | G | G | G |
| ^*^955 | G | A | A | A | A | A |
| 978 | T | C | T | C | C | C |
| 990 | T | G | T | G | G | G |
| 1068 | A | G | G | G | G | G |
| 1159 | C | T | T | T | T | T |
| 1200 | T | T | T | T | C | T |

**^a^** The positions of the polymorphic nucleotides. The first nucleotide of the start codon is 1. +, insertion; *, non-synonymous SNP.
